# Cornelia de Lange Syndrome mutations in SMC1A cause cohesion defects in yeast

**DOI:** 10.1093/genetics/iyad159

**Published:** 2023-08-31

**Authors:** Jingrong Chen, Erin N Floyd, Dean S Dawson, Susannah Rankin

**Affiliations:** Program in Cell Cycle and Cancer Biology, Oklahoma Medical Research Foundation, 825 NE 13th St. Oklahoma City, OK 73104, USA; Program in Cell Cycle and Cancer Biology, Oklahoma Medical Research Foundation, 825 NE 13th St. Oklahoma City, OK 73104, USA; Program in Cell Cycle and Cancer Biology, Oklahoma Medical Research Foundation, 825 NE 13th St. Oklahoma City, OK 73104, USA; Department of Cell Biology, University of Oklahoma Health Sciences Center, Oklahoma City, OK 73104, USA; Program in Cell Cycle and Cancer Biology, Oklahoma Medical Research Foundation, 825 NE 13th St. Oklahoma City, OK 73104, USA; Department of Cell Biology, University of Oklahoma Health Sciences Center, Oklahoma City, OK 73104, USA

**Keywords:** cohesinopathy, disease alleles, SMC1, chromosome segregation, cell cycle, Genetics Models of Rare Diseases

## Abstract

Cornelia de Lange Syndrome (CdLS) is a developmental disorder characterized by limb truncations, craniofacial abnormalities, and cognitive delays. CdLS is caused mainly by mutations in genes encoding subunits or regulators of the cohesin complex. Cohesin plays 2 distinct roles in chromosome dynamics as follows: it promotes looping, organization, and compaction of individual chromosomes, and it holds newly replicated sister chromatids together until cell division. CdLS-associated mutations result in altered gene expression likely by affecting chromosome architecture. Whether CdLS mutations cause phenotypes through impact on sister chromatid cohesion is less clear. Here, we show that CdLS-associated mutations introduced into the SMC1A gene of budding yeast had measurable impacts on sister chromatid cohesion, mitotic progression, and DNA damage sensitivity. These data suggest that sister chromatid cohesion-related defects may contribute to phenotypes seen in CdLS affected individuals.

## Teaser

Mutations in the chromosome cohesion apparatus lead to developmental disorders called cohesinopathies, which have been attributed to changes in transcription. Here, Chen *et al*. exploit sensitive assays in budding yeast to assess the impacts of disease-associated mutations in the highly conserved cohesin subunit SMC1 on chromosome segregation and cell cycle progression. Their data suggests that some of the phenotypes in Cornelia de Lange Syndrome—a cohesinopathy—might also be attributable to cell cycle defects due to compromised sister chromatid cohesion.

## Introduction

Cohesin, a multi-subunit protein complex, was originally characterized for its ability to tether sister chromatids together from the time they are made during DNA replication until chromosome segregation at anaphase (reviewed in Parenti and Kaiser (Morales and Losada 2018)). Cohesion between sister chromatids, also referred to as *trans*-cohesion, promotes their accurate segregation during cell division and is necessary for the efficient repair of certain types of DNA damage. Insufficient inter-sister or *trans*-cohesion can lead to cell cycle arrest and/or mitotic delays due to activation of the spindle checkpoint, a surveillance mechanism that is critically dependent on cohesion between sister chromatids (Sonoda *et al*. 2001; Deehan and Heald 2004).

In addition to its role in chromosome segregation, the cohesin complex also plays a critical role in gene regulation through its ability to promote intrachromosomal or *cis* interactions, particularly in higher eukaryotes. Cohesin promotes the formation of chromatin loops, resulting in chromosome folding and compaction, and thus the formation of topologically associated domains (Gassler *et al*. 2017; Rao *et al*. 2017; Schwarzer *et al*. 2017; Wutz *et al*. 2017), reviewed in Davidson and Peters (2021). The limits of many cohesin-dependent loops are defined by chromatin binding of the transcriptional insulator protein CTCF (Parelho *et al*. 2008; Rubio *et al*. 2008; Stedman *et al*. 2008) (reviewed in Merkenschlager and Nora (2016)). Cohesin can also act with the transcriptional coactivator Mediator to bring certain enhancer-promoter pairs into proximity (Kagey *et al*. 2010), and with CTCF helps to cluster groups of enhancers thereby promoting expression of specific gene sets (Ing-Simmons *et al*. 2015). Consistent with a role for cohesin in gene regulation, the cohesin loader NIPBL was first discovered for the role of its *Drosophila* homolog in developmental gene regulation (Rollins *et al*. 1999) and in mammals heterozygous mutations in the gene result in changes in transcriptional programs (Kawauchi *et al*. 2009). In fact, some genes depend on cohesin for their expression, perhaps due to the role of cohesin in the spatial organization of genes and enhancers (Seitan *et al*. 2013; Sofueva *et al*. 2013; Zuin *et al*. 2014). Despite this strong correlation between cohesin, chromosome topology, and gene regulation, some cohesin inactivation studies suggest that cohesin may primarily play an indirect role in gene regulation, perhaps by controlling enhancer clustering (Ing-Simmons *et al*. 2015; Krivega and Dean 2017; Rao *et al*. 2017; Thiecke *et al*. 2020).

**Fig. 1.**
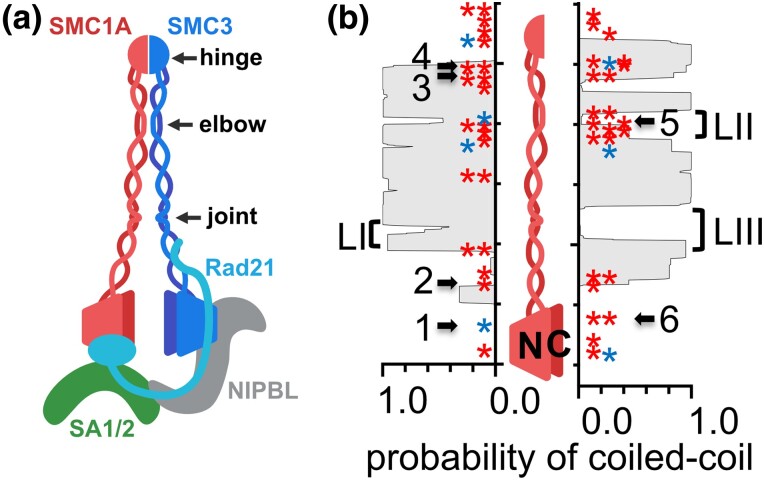
SMC1A mutations used in this study. a) Cartoon of the cohesin complex. Not all subunits are present at all times. PDS5 can interact in the region occupied by NIPBL in the drawing. Cohesin is a dynamic structure that can open at multiple interaction sites and fold over at the elbow such that the hinge is near RAD21/NIPBL (Bürmann *et al*. 2018; Petela *et al*. 2021). b) Locations of the Cornelia de Lange mutations evaluated in this study, numbered 1–6, are shown relative to the estimated positions of coiled-coil domains of human SMC1A protein. Other CdLS-associated missense (red asterisks) or in-frame deletions (blue asterisks) are also shown. Tick marks are at 100 amino acid intervals. Gray shading indicating the probability of coiled-coil formation, calculated using Paircoil2 (McDonnell *et al*. 2006), is plotted along the length of human SMC1A. Alignments of the predicted coiled-coil domains of human SMC1A and budding yeast Smc1 are shown in Supplementary Fig. 1.

The genetic disorder Cornelia de Lange Syndrome (CdLS) is characterized by developmental abnormalities such as limb defects, facial dysmorphism, growth retardation, and cognitive impairments. CdLS (CdLS; OMIM 122470, 300590, 610759, 614701, 300882) is classified as a *cohesinopathy*, a series of related disorders attributable to primarily to mutations in genes that encode subunits or regulators of the cohesin complex. CdLS-causing mutations were first identified in the *NIPBL* gene which encodes the Nipped-B-like protein, a subunit of a dimeric complex that loads cohesin onto chromosomes (Krantz *et al*. 2004; Tonkin *et al*. 2004). Subsequently, CdLS-causing mutations have been identified in other cohesin-related genes, such as *SMC1A*, *SMC3*, and *RAD21*, which encode core cohesin subunits, the *CDCA5* gene encoding cohesion maintenance factor Sororin, and in *HDAC8* which encodes a cohesin deacetylase (Musio *et al*. 2006; Deardorff *et al*. 2007; Deardorff *et al*. 2012; Mannini *et al*. 2013; Kaiser *et al*. 2014; Boyle *et al*. 2015). SMC1A and SMC3 are large, coiled-coil-containing proteins that interact to form a heterodimer that interacts with RAD21 and SA subunits, forming the core cohesin complex (Fig. 1a). Most CdLS-causing mutations in genes encoding subunits of the cohesin complex are missense mutations or small in-frame deletions and result in less severe forms of the disorder (Sarogni *et al*. 2020).

Because cohesin plays multiple critical roles in chromosome structure, function, and segregation, several models have been proposed to explain the phenotypes seen in CdLS. There is compelling evidence that altered gene expression is the central contributing factor to CdLS. CdLS patient-derived cell lines with mutations mapped to NIPBL and SMC1A exhibit altered expression of numerous genes (Liu *et al*. 2009; Izumi *et al*. 2015). In addition, in rare CdLS patients whose mutations do not map cohesion genes, and in patients with syndromes related to CdLS, the causative mutations map to a collection of genes involved in gene regulation or global chromatin functions (reviewed in Parenti and Kaiser (2021)). Together, these data strengthen the conclusion that altered gene regulation is a common contributor to this class of developmental disorders. Consistent with this, in vitro studies of the impact of CdLS-associated mutations in SMC1A and NIBPL show reductions in DNA loop extruding activity (Bauer *et al*. 2021; Panarotto *et al*. 2022). In yeast, cohesin also plays important roles in rDNA condensation and nucleolar function (Lavoie *et al*. 2004; Harris *et al*. 2014). This has led to the proposal that cohesin-dependent changes in the integrity of rDNA repeats, rDNA condensation, and nucleolar structure are causative in cohesinopathies (McNairn and Gerton 2008a; McNairn and Gerton 2008b; Gard *et al*. 2009; Xu *et al*. 2013; Ball *et al*. 2014; Harris *et al*. 2014).

In some cohesinopathies, defects in sister chromatid cohesion present as morphological changes in mitotic chromosomes. For example, in Robert's Syndrome, a cohesinopathy caused by mutations in the gene encoding the cohesin regulator ESCO2, a separation between some sister centromere pairs can be observed in chromosome spreads (Tomkins *et al*. 1979; Vega *et al*. 2005; Gordillo *et al*. 2008; Vega *et al*. 2010). However, consistent defects in *trans*-cohesion have not been reported in CdLS. Some patient-derived cell lines with mutations in the cohesin loader *NIPBL* revealed loss of sister chromatid cohesion phenotypes, but evaluations of patient-derived cell lines with *SMC1A* mutations and lymphocytes from CdLS patients reported no obvious sister cohesion defects (Castronovo *et al*. 2009; Revenkova *et al*. 2009). Similarly, *Drosophila* models heterozygous for mutations in *NIPBL* (as is seen in some CdLS patients) show no obvious chromosome cohesion defects (Rollins *et al*. 2004). Since severe loss in sister chromatid cohesion is lethal (Nasmyth and Haering 2009; Brooker and Berkowitz 2014), it is likely that sister chromatid cohesion defects due to CdLS mutations are too subtle to be detected in assays based on chromosome morphology.

The cohesin complex is highly conserved throughout eukaryotic phylogeny. This allowed us to assess the impact of CdLS mutations on *trans*-cohesin function using the fungal model, *Saccharomyces cerevisiae*. Using both sensitized assays and live cell imaging, we characterized the phenotypes of 6 CdLS mutations in conserved residues in the *SMC1A* protein. Most of the mutations resulted in changes that are consistent with mild defects in *trans*-cohesion, including reduced chromosome segregation fidelity, reduced sister chromatid cohesion, and spindle checkpoint activation resulting in mitotic delays. These data suggest that some phenotypes in affected individuals may be attributable to subtle defects in sister chromatid cohesion.

## Methods

### Strains and primers

The genotypes of the strains used in this work are shown in Supplementary Tables 1 (diploid strains) and 2 (haploid strains). All strains are derived from YNN281. Yeast media, culture techniques, and strain construction methods are as described in Amberg *et al*. (2005).

### Strain construction

Polymerase chain reaction (PCR)-based methods were used to create deletions of open reading frames and mutated versions of *SMC1*. Primers used to build and confirm the strains are shown in Supplementary Tables 3 and 4, respectively. Strains containing mutations in the *SMC1* gene were built by a modified 2-step gene replacement method. Briefly, *SMC1* gene fragments were amplified using high-fidelity phusion (New England Biolabs) polymerase with primer sets (Supplementary Table 3) to generate the appropriate mutations within the gene fragments, which were then cloned into the pRS404 vector (Sikorski and Hieter 1989). The vector was linearized and transformed into the desired yeast strain and colonies were selected on plates lacking tryptophan (the selectable marker in pRS404). After integration of the plasmid was confirmed by PCR, the strains were plated on media containing 5-fluoroanthranilic acid to select against vector sequence (Toyn *et al*. 2000). Loss of vector was confirmed by PCR, and retention of the mutations was screened for by genomic PCR and confirmed by sequencing. For in vivo sister chromatid cohesion assays, chromosome *III* was marked near its centromere (coordinates 113101–113583) by integration of the plasmid OPL509 which carries approximately 256 *lac* operon operator (lacO) repeats (Straight *et al*. 1996). Correct integration was confirmed genetically. GFP-lacI was expressed from the *CYC1* promoter to generate a GFP focus at *CEN3*. For immunoblot assays, the 6HA-KANMX4 gene fragment was PCR amplified from pYM14 (Janke *et al*. 2004) with primer sets (Supplementary Table 3) that target the C terminus of SMC1 gene. The PCR product was transformed into the desired yeast strain, and colonies were selected on plates containing G418. The integration of the cassette was confirmed by PCR.

### Western blot analysis of SMC1 expression

Total protein extracts were prepared by TCA precipitation (Foiani *et al*. 1994). Briefly, 5 × 10^7^/ml cells in logarithmic growth were collected and washed once with water and resuspended in 16.6% trichloroacetic acid at room temperature. After addition of the same volume of 0.5-mm zirconia/silica beads (Biospec Products), cells were disrupted by a Bullet Blender for 5 minutes. Beads were washed once with 100 µl of 16.6% trichloroacetic acid, and the resulting extract was spun for 5 minutes at 13,000 rpm at 4°C. The pellet was washed once with acetone and resuspended in 100 µl of SDS-PAGE sample buffer, pH neutralized by addition of 1-M Tris base, boiled for 5 minutes, and clarified by centrifugation. Proteins were separated by SDS-PAGE and detected by immunoblotting, using monoclonal antibodies against HA (C29F4, Cell Signaling) and PGK (22C5, Molecular Probes).

### Chromosome segregation assay

Strains were grown in YPAD (yeast extract, peptone, adenine, dextrose) medium at 30°C, except DJC5−. This strain was grown in a medium to select for cells that carried the mini-chromosome used to assay mis-segregation. Cells were diluted and plated on a synthetic complete medium supplemented with 6-μg/ml adenine. This level of adenine allows growth of the *ade2-101* yeast cells that have lost the *SUP11* gene marker and permits accumulation of the red pigment used to identify segregation errors. Plates were incubated at 30°C until colonies were large (3–4 days) and further stored at 4°C for a few days to enhance differentiation of the color phenotypes. The half-sectoring assay was scored as previously described (Hieter *et al*. 1985).

### Cohesion assay

Asynchronous cells were grown to mid-log phase at 30°C in YPAD media, then pelleted and washed with H_2_O. Then cells were resuspended at 10^−6^/ml in a complete medium (Sunrise Science Products Cat. 1729-500) with 3 × 10^−6^ M α factor (GenScript RP01002) and incubated at 23°C for 3 hours. G1 arrested cells were washed 3 times in YPAD with 0.1-mg/ml Pronase E (Sigma P6911), followed by 1 more wash in YPAD. Cells were then resuspended at 5 × 10^6^/ml in YPAD containing 1% DMSO and 15-µg/ml nocodazole (Sigma) and incubated at 23°C for 3 hours to arrest in M phase. A total of 7.5-µg/ml nocodazole was re-added at 1.5 hours. Samples were collected at 0 and 3 hours following addition of nocodazole for scoring. In large-budded cells with 1 DAPI mass and elongated, or separated red dots that were less than 1 µm apart, we scored the number of GFP dots in each DAPI mass. We then plotted the percentage of cells with 2 GFP dots. At least 100 cells were counted in each replicate. Three or more replicates were performed for each strain.

### Mitotic delay assay

G1 arrest was achieved as for cohesion defect assay, except that cells were grown in 30°C. G1 arrested cells were washed 3 times in a complete medium (Sunrise Science Products Cat. 1729-500) with 0.1-mg/ml Pronase E (Sigma P6911), followed by washing once in a complete medium. Cells were then resuspended at 5 × 10^6^/ml in a complete medium and incubated in 30°C for 3 hours in CellASIC microfluidics plates (Millipore) for live cell imaging. Spc42-RFP allowed visualization of spindle pole bodies. The time interval between when the single RFP focus split into 2 foci (spindle formation) and when the 2 foci dramatically increased in their separation (anaphase onset) was measured and plotted.

### X-ray irradiation sensitivity test

Asynchronous cells were grown to mid-log phase at 30°C in YPAD media, collected by brief centrifugation, washed with water, and resuspended in the original volume of water. The cells were exposed to an RS-2000 source for the designated dosages. Appropriate dilutions of cells were plated on YPAD agar plates and incubated for 2 days. Each experiment was performed 3 times. The average of 3 replicate platings was plotted.

### rDNA condensation assay

Log-phase cells expressing the rDNA-binding protein Net1 fused to GFP (Net1-GFP) were suspended at 1 × 10^6^/ml in a SD Sunrise medium (Sunrise Science Products) containing 3 × 10^6^ M α factor, for 2 hours at 30°C. The cells were then washed with 3 times with YPAD containing 0.1-mg/ml pronase E (Sigma), followed by 1 wash with YPAD. Cells were then resuspended in YPD containing 1% DMSO at 4 × 10^6^/ml. After 1 hour of incubation at 30°C, rDNA condensation was analyzed in cells with large buds (daughter cell diameter greater than one-half of mother cell diameter) that contained 1 DAPI staining mass by visualizing Net1-GFP. Cells were placed in 1 of 4 categories based on Net-GFP morphology as described previously (Lavoie *et al*. 2004). The 4 categories were as follows: puff/amorphous (a single large amorphous cap on the edge of the nucleus larger in diameter than one-fourth the diameter of the DAPI staining mass), cluster (a GFP signal over-lapping the nucleus, smaller than one quarter the diameter of the DAPI staining mass), loop (condensed arc shape protruding from the DAPI staining mass), or line (a single line of GFP signal extending out from the DAPI mass).

### Statistics and sequence analysis

Graphing and statistical analysis were done using Prism software and web-based tools (QuickCalcs), both from Graphpad (La Jolla, CA). Protein alignments and sequence logos were generated using the Clustal Omega algorithm in Geneious Prime (Biomatters, Auckland, New Zealand). Parallel coiled-coil folds were predicted using Paircoil2 (McDonnell *et al*. 2006).

### Microscopy

rDNA assay images were collected using a Zeiss AxioImager microscope with band-pass emission filters, a Roper HQ2 charge coupled device, and AxioVision software. Mitotic delay experiments (every 3 minutes for 3 hours) were performed with Onix2 microfluidics system (Millipore) using Y04C-02 plates with a flow rate of 2 pounds per square inch. Images were collected with a Nikon Ti2 inverted microscope equipped with the Perfect Focus system, an ORCA FLASH camera, automated stage, a Lumencor LED light source, and NIS software. Images were processed and analyzed using Nikon NIS Elements software. Sister chromatin cohesion assay images were also collected with the Nikon Ti2 inverted microscope system described above, and images were processed using Nikon NIS Elements software.

## Results

A number of mutations that cause Cornelia de Lange Syndrome [*CDLS2* (MIM: 300590)] have been mapped to the gene encoding the core cohesin subunit, *SMC1A*, which encodes the SMC1A protein (Musio *et al*. 2006; Borck *et al*. 2007; Deardorff *et al*. 2007). SMC1 proteins have a well-conserved structure from yeast (Smc1) to humans (SMC1A). The protein folds over on itself at a globular region near its midpoint (the hinge), and the N and C termini come together to form a second globular domain, called the head (Fig. 1a). The SMC3 protein is similarly folded, and SMC1A and SMC3 interact at both the hinge region and at their head domains, which form a pair of ATP-binding sites (Fig. 1a). The head domains also interact with the RAD21 subunit (Scc1 in yeast), and SA1 or SA2 (Scc3 in yeast) to form a tetramer. Chromatin-bound cohesin also interacts with a regulatory subunit called PDS5. The long coiled-coils in SMC family proteins are predicted to be interrupted by short regions that extend from the coiled-coils as loops, designated LI, LII, and LIII (Fig. 1b and Supplementary Fig. 1) (Beasley *et al*. 2002). More recently, these regions have been referred to as the elbow (amino acids 355–410 and 780–815 in human SMC1A) and joint (amino acids 205–255 and 940–1000) regions (Bürmann *et al*. 2018). The SMC1A and SMC3 proteins can fold over at the elbow regions bringing the hinge in proximity of the joint. In the folded configuration, the SMC1A/3 hinge interacts with NIPBL (called Scc2 in yeast) or PDS5, thereby affecting cohesin loading or DNA loop extrusion, respectively (Bürmann *et al*. 2018; Petela *et al*. 2021). Consistent with this, in budding yeast, mutations in Smc1 moiety of the hinge (*smc1-D588Y*) impact cohesin-Scc2 interactions and mutations in the joint region (*smc1-209L1-2*) allow cohesin loading but ablate sister chromatid cohesion (Milutinovich *et al*. 2007; Petela *et al*. 2021).

**Fig. 2.**
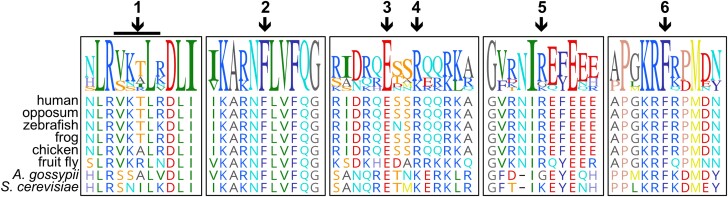
The sequence context of the human *SMC1A* CdLS mutations from this study. The positions of residues for the 6 mutations evaluated here (V58-R62del, F133V, E493A, R496A, R790Q, F1123L) are indicated with black arrows. The black bar indicates the in-frame deletion of the first mutation. Clustal Omega alignments of the relevant sequences in the human protein with Smc1 proteins from diverse eukaryotic organisms are shown. The strength of the alignment is indicated by the height of the colored letters at the top.

The positions of CdLS-associated *SMC1A* missense and deletion mutations reported in the Human Genome Mutation Database (http://www.hgmd.cf.ac.uk/ac/index.php) are shown in Fig. 1b. We sought to explore the ways in which CdLS mutations might disrupt cohesin function by exploiting simple quantitative assays for many aspects of mainly *trans*-cohesion function that can be performed in yeast. We chose for analysis 6 mutations that were previously characterized and are associated with CdLS phenotypes of varying severity (Table 1) (Deardorff *et al*. 2007). The CdLS mutations selected for this analysis were among those that lie in conserved sequence blocks and affect amino acids that are similar or identical in the human and yeast proteins (Fig. 2). Importantly, not all CdLS mutations alter amino acids that are conserved between humans and yeast, and these mutations could impact functions that are not conserved in yeast. For each of the chosen mutations, we created the same mutation in the yeast *SMC1* gene at its endogenous locus.

**Table 1.** Mutations in SMC1A assessed in this study.

Mutation #	Human mutation	Phenotype	Gender of patient	Yeast mutation
1	V58-R62del	Mild	F	S59-K63del
2	F133V	Mild	M	F147V
3	E493A	Moderate to severe	M	E508A
4	R496H	Mild	F	K511H
5	R790Q	Mild	F	K801Q
6	F1122L	Mild	F	F1123L

### Some conserved CdLS-associated mutation in SMC1 disrupt rDNA packaging

**Fig. 3.**
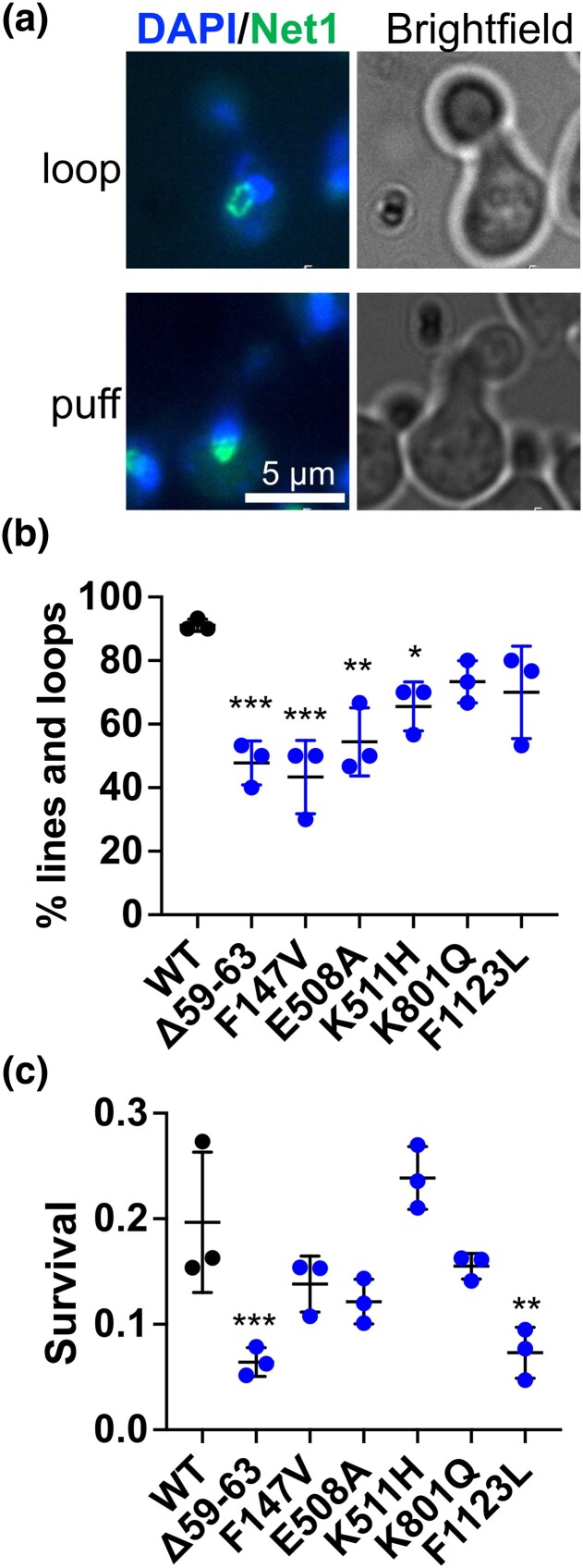
Nucleolar condensation and DNA damage repair phenotypes of yeast strains with conserved CdLS-associated mutations. a) Nucleolar condensation was monitored by imaging a GFP-tagged version of Net1. Cells were collected for imaging 1 hour after release from a G1 arrest. Cells with large buds (greater than one-half the diameter of the mother cell—consistent with being in mitosis) were scored for nucleolar morphology (blue, DAPI staining; green, GFP). Most wild-type cells had condensed lines or loops of Net1-GFP signal (top panel). Others had amorphous puffs of Net1-GFP staining (bottom panel). b) Graph showing percent of scored cells with loop or line morphology. Each point represents the average of thirty cells scored in 1 biological replicate. c) Radiation sensitivity phenotypes of conserved CdLS-associated mutations. Cells in mid-log phase were exposed to 600 Gy of X-irradiation then plated for growth on a rich medium. The fraction of surviving cells relative to nonirradiated controls is indicted in the graph. Each point represents 1 biological replicate. For both experiments, a 1-way ANOVA statistical analysis was performed (*P < 0.05, **P < 0.01, ***P < 0.001).

A previous study that used budding yeast to evaluate conserved CdLS mutations in cohesin and cohesin-regulatory genes *ECO1* (W216G), *SMC1* (E508A; Q843Δ), and *SCC2* (R716L; D730V; G1242R) concluded that these mutations did not result in cohesion defects, but 2 of the alleles (*eco1-W216G* and *scc2-D730V*) resulted in altered nucleolar morphology and condensation (Gard *et al*. 2009), while others have shown loss of cohesion in the case of Eco1 W216G (Borrie *et al*. 2017) We therefore tested for these defects in strains bearing CdLS-associated *SMC1* mutations. Cells expressing Net1-GFP were imaged to reveal nucleolar morphology. In wild-type cells, the rDNA in most cells condenses to form a tight loop or line that can be visualized by the associated Net1-GFP (Lavoie *et al*. 2004). Cells were arrested in G1 with alpha factor then released into the cell cycle. Mitotic cells (large bud, single DAPI-staining nuclear mass) were imaged. Approximately 95% of wild-type control cells exhibited condensed lines or loops of rDNA. In contrast, 4 of the 6 mutants exhibited elevated levels of the less condensed rDNA that manifests as a puff-like Net1-GFP morphology (Fig. 3, a and b). Therefore, like the previously characterized *eco1-W216G* and *scc2-D730V* CdLS alleles, these *SMC1* CdLS mutations affect rDNA behavior.

### Some conserved CdLS-associated mutations in SMC1 result in sensitivity to radiation-induced damage

Cohesin is critical for the timely repair of DNA double-strand breaks, and cell lines derived from CdLS patients carrying mutations in *NIPBL* (*SCC2* in budding yeast), *SMC1A*, and *SMC3* exhibit sensitivity to ionizing radiation (Vrouwe *et al*. 2007; Revenkova *et al*. 2009). To test whether CdLS *SMC1A* mutations affect this cohesin function, we monitored the sensitivity to ionizing radiation of strains bearing the conserved CdLS mutations. Cells in logarithmic growth were subject to 600 Gy of radiation and then plated to assess survival. Two of the 6 mutations (*smc1-Δ59-63* and *smc1-F1123L*) conferred significant sensitivity to ionizing radiation (Fig. 3c). This finding corresponds to results in a study of CdLS patient-derived cells (Revenkova *et al*. 2009), in which cell lines with cognates of the 2 radiation-sensitive mutations in yeast (*SMC1A-ΔV58-R62* and *SMC1A-F1122L*) similarly exhibit sensitivity to ionizing radiation. In contrast, patient-derived cells with the *SMC1A-R496H* mutation, like yeast with the homologous *smc1-K511H* mutation, did not exhibit radiation sensitivity. Thus, in this assay, the yeast and human mutations phenocopy each other.

### Some CdLS-associated mutations in yeast SMC1 result in defective chromosome segregation

**Fig. 4.**
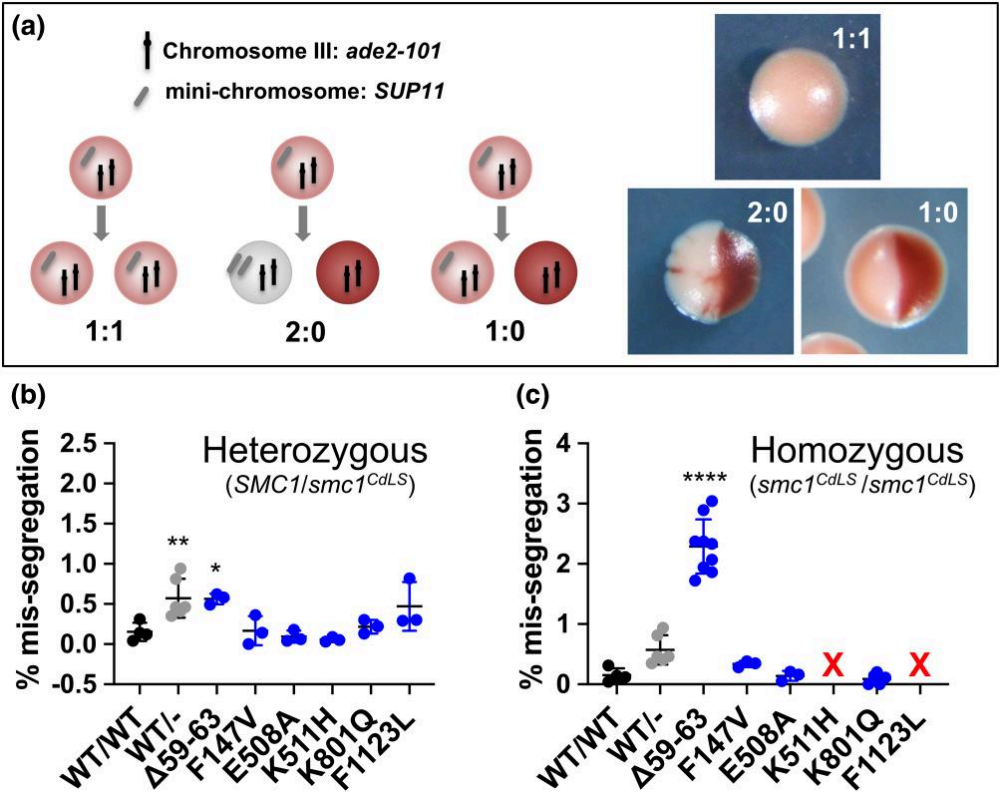
Chromosome mis-segregation in yeast strains carrying conserved CdLS-associated mutations. a) Illustration of the sensitized mini-chromosome segregation assay. A marker mini-chromosome encodes a suppressor tRNA (*SUP11*), which suppresses the ocher nonsense mutation in an *ade2-101* allele carried on natural chromosome *III* (Hieter *et al*. 1985). In the absence of suppression, *ade2-101* cells accumulate a pigmented intermediate in the adenine biosynthetic pathway, resulting in red cells and colonies. The copy number ratio between the marker mini-chromosome and the *ade2-101* allele determines the degree of suppression, and thus the colony color. Full suppression (1:1 ratio mini-chromosome:Chr *III*) results in white colonies, while partial suppression (1:2 ratio, mini-chromosome:Chr *III*) results in pink colonies, and in the absence of suppression (no mini-chromosome), colonies are red. Colonies are scored based on the first segregation event (half colony only), and smaller sectors that arise later during outgrowth are ignored. Examples of sectored colonies are shown. b) Chromosome mis-segregation rates of strains with heterozygous CdLS mutations (*SMC1/smc1^CdLS^*). Shown are the percent of colonies exhibiting mis-segregation of the marker chromosome in the first cell division after plating. The total frequency of chromosome mis-segregation events (2:0 segregation and 1:0 segregation) is reported. Symbols: black, wild-type homozygous; gray, wild-type hemizygous; blue, CdLS-associated mutations. c) Chromosome mis-segregation rates of strains with homozygous CdLS mutations (*smc1^CdLS^/smc1^CdLS^*). Red “X” = strains that were not scorable due to extreme instability of the marker chromosome. A statistical analysis was performed with a 1-way ANOVA (*P < 0.05, **P < 0.01, ****P < 0.0001).

Prior studies have shown that alterations in gene expression are likely the primary reason that mutations in cohesin-related genes lead to CdLS (Dorsett and Krantz 2009; Liu *et al*. 2009). In contrast, assays in various CdLS models have not consistently revealed defects in sister chromatid cohesion. However, most of these studies assayed for evidence of profound loss of sister chromatid cohesion—for example frequent and complete separation of sister chromatids in chromosome spreads (Kaur *et al*. 2005; Castronovo *et al*. 2009; Revenkova *et al*. 2009). These studies made clear that gross sister chromatid cohesion failure is a poor diagnostic tool for CdLS (Castronovo *et al*. 2009). However, those assays were not designed to identify milder defects in sister chromatid cohesion that would be compatible with cell proliferation and organism survival. Indeed, the 6 conserved CdLS mutations evaluated here all support normal growth of budding yeast under non-stressed conditions (Supplementary Fig. 2a), demonstrating that in cells with these mutations, all of the chromosomes segregate correctly in most cell cycles. The mutations did not significantly affect protein expression levels (Supplementary Fig. 2b). Here, we asked whether conserved CdLS-associated mutations in yeast *SMC1* might result in mis-segregation of 1 or a few chromosomes in occasional cells, or perhaps put cells under stress during mitosis. To test for this, we used an assay that detects the mis-segregation of a single, truncated, nonessential reporter chromosome (or mini-chromosome). The small size of the test chromosome renders it more sensitive than natural chromosomes to defects in segregation machinery, and thus facilitates the evaluation of mutations that diminish, but do not abolish, segregation fidelity (Hegemann *et al*. 1988). Mis-segregation of the mini-chromosome provides a colorimetric read-out because the mini-chromosome carries a suppressor of the *ade2-101* mutation that causes accumulation of a red pigment (Fig. 4a) (Hieter *et al*. 1985). The suppressor mutation on the mini-chromosome affects accumulation of the pigment in a dose dependent manner allowing cells that have lost the mini-chromosome, or gained extra copies, to be detected by colony color (Fig. 4a). Because the mini-chromosome carries no essential genes, cells that lose it continue to propagate and can be scored in the colony color assay. In diploid cells, the system allows the detection of both mis-segregation, in which 2 copies of the chromosome segregate to 1 daughter cell and none segregate to the other (2:0 segregation), and chromosome loss, in which 1 copy of the marker chromosome is lost during cell division (1:0 segregation) (Fig. 4a). Chromosome loss may occur when 1 chromatid, which has lost its association with its sister, does not attach to microtubules from either side of the spindle, and is left in the spindle mid-zone at anaphase I, and fails to be included in either daughter nucleus (Hieter *et al*. 1985). Both types of errors occur when sister chromatid cohesion fails. Thus, this system provides a sensitive assay for defects in establishing or maintaining sister chromatid or *trans*-cohesion.

Because CdLS-associated *SMC1A* mutations occur mainly in a heterozygous configuration in females and are present as the sole allele of *SMC1A* in males, we scored the chromosome mis-segregation phenotypes of the alleles in both heterozygous and homozygous configurations. When screening the yeast *SMC1* alleles in the heterozygous configuration, we compared their behavior to both the wild-type strain (*SMC1/SMC1*) and a heterozygous strain, in which 1 copy of *SMC1* was deleted from the diploid background (*SMC1*/*smc1Δ*). The *SMC1/smc1Δ* control showed significantly higher chromosome segregation error rates than the wild-type control demonstrating that the *SMC1* is haploinsufficient (Fig. 4a). Earlier work in budding yeast has shown that levels of the Scc1/Mcd1 subunit of cohesin can be lowered several fold with no clear impacts on sister chromatid cohesion, although these investigators used different, and probably less sensitive, assays to score cohesin function (Heidinger-Pauli *et al*. 2010). In the heterozygous configuration, chromosome mis-segregation was significantly disrupted by only 1 of the CdLS-associated alleles (*smc1-Δ59-63*) (Fig. 4b and Supplementary Table 2). Because Smc1 is limiting in this assay, an elevated mis-segregation phenotype in a heterozygote could reflect insufficient Smc1 function rather than dominant negative action of the mutant allele. Because none of the CdLS heterozygotes showed a greater defect than the *SMC1/smc1Δ* control, there is no evidence that any of them have dominant-negative phenotypes in this assay ^57^(Fig. 4b).

Five of the CdLS-associated mutations had no significant effect on chromosome segregation when present with a wild-type copy of *SMC1* in the cell. Since these heterozygotes (*SMC1/smc1-CdLS*) exhibited better segregation fidelity than the *SMC1/smc1Δ* control and indistinguishable from the *SMC1/SMC1* control, these mutations must be supplying at least partial function, such that in the presence of a wild-type copy the cells can segregate the test chromosome accurately. To test whether the CdLS-associated mutations lost at least some ability to contribute to chromosome segregation, we re-screened the alleles in the homozygous configuration in diploids (Fig. 4 and Supplementary Table 3). For 2 of the mutants the mini-chromosome was lost so frequently that it was not possible to score the sectoring phenotype (Fig. 4c alleles marked by a red “X”). In addition, the *smc1-Δ59-63* allele also exhibited severe defects in the homozygous configuration.

A previous sister chromatid cohesion assay of CdLS patient-derived cell lines showed that the human cognate mutations of *smc1-Δ59-63*, *smc1-K511H*, and *smc1-F1123L* had no cohesion defects in an assay in which separation of greater than 50% of the sister chromatid pairs in each chromosome spread was scored as loss-of-cohesion (Revenkova *et al*. 2009). Loss of sister chromatid cohesion at this level is likely to render the yeast strains inviable. Thus, as in the human cells, the CdLS mutations do not catastrophically compromise chromosome segregation in yeast, but the sensitized assay reveals that most of the mutations diminish the ability of Smc1 protein to ensure high fidelity chromosome segregation (Fig. 4a).

### CdLS mutations in *SMC1* cause cohesion defects

**Fig. 5.**
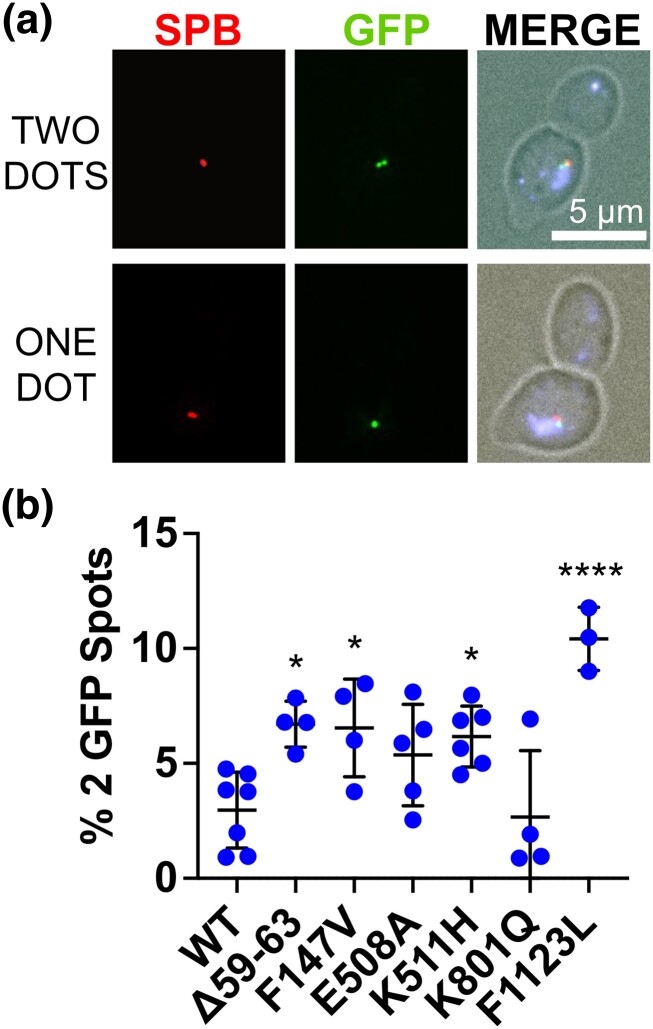
Cohesion failure in yeast cells with conserved CdLS-associated mutations. a) Sister chromatid cohesion was scored in a strain with an array of *lac* operator repeats inserted adjacent to the centromere and expressing a GFP-lacI fusion protein. The spindle pole body is tagged by expression of an Spc42-DsRed fusion protein. Haploid cells were arrested with alpha factor in G1 then released into a medium with nocodazole to prevent spindle formation. Cells with large buds and 2 Spc42-dsRed foci were scored for separation of GFP spots. a) Examples of cells with 2 (top panel) and 1 (bottom panel) GFP spots. b) The percent cells with 2 GFP spots for wild-type controls and strains with CdLS-associated mutations. Each point represents 1 biological replicate of 100-to-140 cells. A statistical analysis was performed with a 1-way ANOVA (*P < 0.05, ****P < 0.0001).

In both human and yeast cells with *SMC1* CdLS mutations, chromosomes segregate correctly in most mitoses. However, the results of the sensitized segregation assay (Fig. 4) demonstrate that these mutations compromise the ability of *SMC1* to ensure high fidelity chromosome segregation. We therefore performed an assay of yeast strains with CdLS-associated mutations that would allow us to detect mild defects in sister chromatid cohesion. To do this, we tagged a natural chromosome near its centromere with a fluorescent protein, GFP-LacI, that binds to an array of *lac* operon operator sequence repeats, making a green dot (Straight *et al*. 1996) (Fig. 5a). These cells were also engineered to express Spc42-DSRed to mark the microtubule organizing center (the spindle pole body: SPB). To perform the assay, haploid cells were synchronized in G1 using alpha factor and released into the cell cycle in the presence of the microtubule depolymerizing agent nocodazole. This treatment causes the cells to arrest in metaphase with a collapsed spindle (yielding side-by-side SPBs). Cells in M phase were identified as those with large buds and a single nucleus. In cells with functional cohesion, the GFP foci on the cohered sisters appear as a single dot in most metaphase cells, whereas reduced cohesion allows the sister chromatids to separate, and 2 dots can be resolved (Fig. 5a) (Michaelis *et al*. 1997). In the wild-type control (*SMC1*), 2.97% of the chromosomes exhibited 2 GFP dots (indicating separation of the sister chromatids). In contrast, 4 of the 6 CdLS mutations resulted in significantly elevated levels of separated sister chromatids (Fig. 5b). These 4 mutants (*smc1-Δ59-63*, *smc1-F147V*, *smc1-K511H*, and *smc1-F1123L*) were also the 4 that showed the highest error rates in the chromosome mis-segregation assay. We conclude that CdLS-associated mutations result in weakly compromised sister chromatid cohesion which is likely the cause of mitotic errors in the sensitized chromosome segregation assay (Fig. 4).

### CdLS-associated mutations in budding yeast *SMC1* cause mitotic delays

**Fig. 6.**
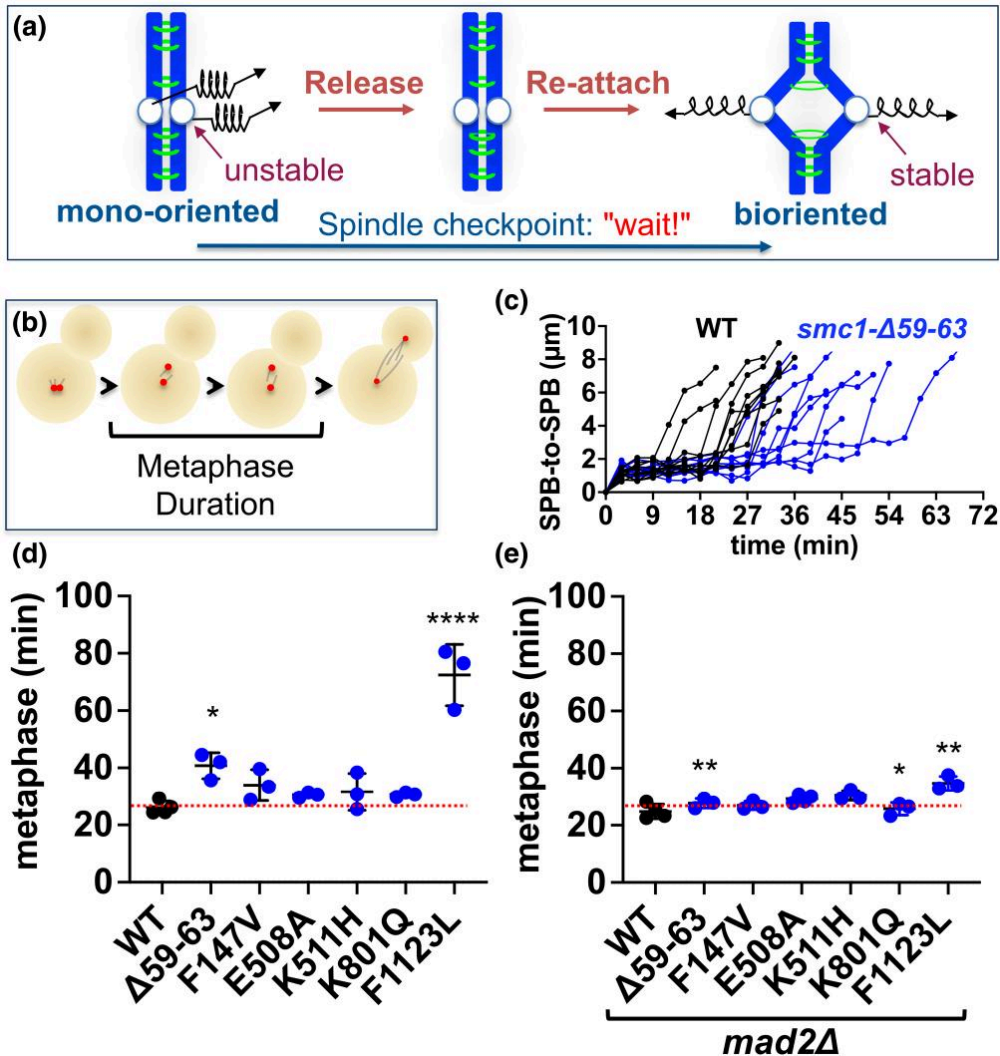
CdLS-associated mutations in yeast *SMC1* cause delays in cell cycle progression. Haploid cells expressing Spc42-DsRed were arrested in G1 using alpha factor then released into the cell cycle. Cells were mounted in a microfluidics chamber and imaged every 3 minutes. The time between spindle formation (the first frame with 2 red foci) and anaphase (the inflection point when spindle length rapidly increases) was measured. a) Cohesin is needed for correct attachment of sister chromatids to the mitotic spindle. b) Cartoon of cells proceeding through metaphase (imaging allowed visualization only of cell shape and the DsRed signal). c) Traces of the distances between the Spc42-DsRed foci in the first 10 individual wild-type cells and *smc1-Δ59-63* cells scored in 1 biological replicate. d) Metaphase duration for wild-type controls and strains with CdLS-associated mutations in *SMC1*. Each point represents the average metaphase duration for the cells measured in 1 biological replicate. A statistical analysis was performed with a 1-way ANOVA (*P < 0.05, ****P < 0.0001). The red dotted line in panels d and e corresponds to metaphase duration of the wild-type control in panel d, to allow comparisons between the 2 panels. e) Metaphase duration for controls and strains with CdLS-associated mutations in *SMC1*, all deleted for the *MAD2* spindle checkpoint gene. Each point represents 1 biological replicate. For each *SMC1* mutant the difference in timing, with and without the *MAD2* deletion was compared with Student's t-test (*P < 0.05, **P < 0.01). The numbers of biological replicates, the number of cells measured in each replicate, and the metaphase duration for each individual cell measured are shown in Supplementary Fig. 3.

A total loss of sister chromatid cohesion as would be caused, for example, by complete inactivation of *SMC1*, leads to failed mitoses and is incompatible with cell or organismal viability ^49,50^. Four of the CdLS-associated mutations evaluated here cause detectable cohesion defects in 1 chromosome (chromosome *III*) in a small percentage of cells (Fig. 5). Assuming chromosome *III* is representative of all 16 chromosomes, then, most cells would be expected to have at least 1 chromosome with compromised cohesion in every mitosis. The high viability of these cells suggests that chromosome segregation is robust. However, reduced cohesion might yet affect cell cycle progression by activation of a checkpoint. The process of correctly attaching sister chromatids to the mitotic spindle requires functional centromeric cohesion (Fig. 6a). Microtubules attach and detach from kinetochores until they reach a stable bi-oriented configuration. Correct attachments are stabilized by tension that is critically dependent on centromeric cohesion between sister chromatids. The spindle checkpoint prolongs metaphase until all sister chromatid pairs are bi-oriented on the spindle. To determine whether the mild cohesion defects caused by the CdLS mutations resulted in spindle checkpoint activation, we assayed our strains for delays in metaphase using live cell imaging. Cells were propagated and imaged in a microfluidics chamber. The SPBs were marked by Spc42-DSRed and images were acquired every 3 minutes to track spindle growth. Cells were scored as entering mitosis in the first imaging frame with 2 SPBs—indicative of spindle formation (Fig. 6b). Entry into anaphase was marked by a rapid increase in spindle length (Fig. 6b). Examples of 10 wild-type and 10 mutant cells (*smc1-Δ59-63*) from 1 replicate of the experiment are shown in Fig. 6c. Wild-type cells spent an average of 26 minutes in metaphase (Fig. 6d and Supplementary Fig. 3). All the mutants exhibited longer than average metaphase durations, with 2 rising to the level of significance (*smc1-Δ59-63* and *smc1-F1123L*). To determine whether the delays due to activation of the spindle checkpoint, we tested whether ablating the spindle checkpoint shortened the delay by deleting the *MAD2* checkpoint gene from our strains. Metaphase timing for the wild-type strain was not affected by the *MAD2* deletion consistent with published observations (Li and Murray 1991). In contrast, all 6 mutant strains proceeded more quickly through metaphase in the *mad2Δ* background, 3 rising to the level of statistical significance (Fig. 6e and Supplementary Fig. 3).

## Discussion

The precise ways in which cohesin gene mutations lead to CdLS phenotypes are not known, but as described in the *Introduction*, there is compelling evidence that altered gene expression is a central contributing factor.

Here, we have performed a series of assays in yeast to determine whether CdLS-associated mutations in *SMC1A* might also impact functions associated with *trans*-cohesion. We have not assayed these collection mutations for their effects on genome-wide transcription profiles. However, the human version of one of them (*smc1-Δ59-63*) was recently analyzed in an in vitro chromatin loop-extrusion assay and exhibited quantifiable defects in this activity as well (Bauer *et al*. 2021). The compelling evidence that CdLS mutations cause transcriptional defects suggests that the mutations we have analyzed are also likely to be defective in transcription control.

Our findings, summarized in Table 2, demonstrate that CdLS mutations impact cohesin's role in multiple aspects of chromatin organization. Our analysis of 6 CdLS-associated mutations in conserved amino acids of *SMC1* reveals that all but one (*smc1-K801Q*) exhibit quantifiable deficiencies in 1 or more processes associated with cohesin function, many of which rely at least in part on *trans*-cohesion (DNA repair, sister chromatid cohesion, metaphase progression, and chromosome segregation), although K801Q had a subtle checkpoint-mediated mitotic delay. Previous assays of the effects of CdLS mutations on *trans*-cohesion monitored more substantial sister chromatid cohesion defects than the assays used here and found no clear defects (Castronovo *et al*. 2009; Revenkova *et al*. 2009). Here, we find that several of the CdLS-associated *SMC1A* mutations that we evaluated cause low levels of chromosome segregation errors, which is consistent with the compatibility of these mutations with life. Importantly, although the sister chromatid cohesion defects associated with the *SMC1A* mutations were mild, some of the mutations appear to cause metaphase delays in many or even most cell cycles. Defective sister chromatid cohesion at centromeres or adjacent sites triggers spindle checkpoint-mediated delays in metaphase (Skibbens *et al*. 1999; Toyoda *et al*. 2002; Toyoda and Yanagida 2006). This is because stabilization of correct microtubule-kinetochore attachments depends on the transmission of tension between the bi-oriented kinetochores, and this requires sister chromatid cohesion at the centromeres. Thus, although the *SMC1A* mutations do not result in total failed sister chromatid cohesion, we suggest that 1 or a few chromosomes in most cells have deficiencies in generating tension at the kinetochores during metaphase, which then trigger spindle checkpoint-mediated cell cycle delays. These delays in budding yeast cell divisions must not be associated with frequent chromosome segregation errors since the strains exhibit largely normal growth.

**Table 2.** Summary: phenotypes conferred by CdLS SMC1 alleles in budding yeast assays.

Mutation #	Allele (yeast)	Defective rDNA condensation?	X-ray sensitivity?	Chromosome loss?	Cohesion loss?	Checkpoint-mediated delay?
1	S59-K63del	✓	✓	✓	✓	✓
2	F147V	✓	—	—	✓	—
3	E508A	✓	—	—	—	—
4	K511H	✓	—	✓ (homozygote)	✓	—
5	K801Q	—	—	—	—	✓
6	F1123L	—	✓	✓ (homozygote)	✓	✓

In our work, we did not see a clear correlation between the severity of the phenotypes in human patients (Tables 1 and 2) and the assays shown here. The only allele tested that causes a severe phenotype in humans was E493A (E508A in yeast), which only showed a defect in rDNA condensation in the yeast assays. We do note that the CdLS-associated alleles tested here had different impacts in the various assays we performed, consistent with that fact that the different mutations probably affect distinct cohesin interactions, conformations, or activities. Recent in vitro studies showed that charge-altering CdLS mutations in the coiled-coil regions of SMC1A (Fig. 1), for example R790Q (yeast K801Q in our study), diminish SMC1A–SMC3 coiled-coil interactions (the rod conformation) and result in reduced loop extrusion but that these mutations do not seem to impact ATP hydrolysis (Bauer *et al*. 2021). In contrast, the Δ58-62 mutation (yeast Δ59-63 in our study) removes surface amino acids in the head domain that promote DNA binding. The Δ58-62 mutant shows slightly reduced stimulation of ATP hydrolysis by DNA binding and slightly reduced loop extrusion. Finally, the F1122L mutation maps immediately adjacent to one of the ATP-binding pockets and likely directly impacts the ATPase function (Marcos-Alcalde *et al*. 2017). Thus, CdLS mutations directly reduce distinct and different molecular actions of cohesin. Combined with our results, it appears that the different functional roles of cohesin (sister chromatid cohesion, chromatin organization and compaction, DNA repair) are differentially impacted when specific molecular activities, such as DNA binding, coiled-coil interactions, or ATPase activity, are reduced.

The 6 mutations we evaluated showed a range of mutant behaviors (Table 2). The K801Q showed no significant defects. Defects in rDNA condensation were exhibited by the other 5, even those with few other defects (F147V and E508A) suggesting that the rDNA condensation assay is perhaps the most sensitive to subtle loss of cohesin activity. It is also possible that the role of cohesin in rDNA condensation in yeast is somehow related to its role in gene expression in human cells (e.g. chromatin loop formation). Chromosome segregation K511H and F1123L, when homozygous, resulted in extreme instability of the marker mini-chromosome. Because the mini-chromosome is so short, it may be especially reliant on centromeric cohesion. It may be that K511, which is not near an ATP-binding site, and F1123 (which is) both contribute to molecular activities that are critical for establishing *trans*-cohesion and had significant impact in this assay. Finally, it is also possible that several of the alleles would show statistically significant defects in multiple assays with larger sample sizes. For example, in the chromosome loss and metaphase delay assays, all the alleles trended toward defective behavior but only a subset were significantly different from wild type. It is also possible that some of the assays we chose are not sensitive enough to detect very subtle loss of function.

In metazoans, spindle checkpoint-mediated delays often trigger apoptotic cell death (Ruan *et al*. 2019). A recent study in *Drosophila* has shown that mitotic delays triggered by sister chromatid cohesion defects result in developmental defects (Silva *et al*. 2018). Here, inactivation of a gene (*san*) that results in modest defects in sister chromatid cohesion caused mitotic delays and severe defects in wing development. These phenotypes could be by-passed by mutations that ablate the spindle checkpoint (*mad2* or *mps1*)—suggesting that the observed developmental defects were attributable to extended metaphase delays. These findings, together with our results, suggest that some CdLS mutations might lead to deficiencies in populating tissues during the appropriate developmental windows due to their triggering of mitotic delays. Thus, although altered gene expression may be the major cause of CdLS, it may be that subtle deficiencies in *trans*-cohesion or other cohesin functions contribute to developmental outcomes in CdLS patients.

## Supplementary Material

iyad159_Supplementary_Data

## Data Availability

All strains and plasmids are available upon request. The authors affirm that all data necessary for confirming the conclusions of the article are present within the article, figures, and tables. Supplemental material available at GENETICS online.
