# Supplementary material for: Cornelia de Lange Syndrome mutations in SMC1A cause cohesion defects in yeast
Source: Genetics. 2023 Aug 31;225(2):iyad159. doi: 10.1093/genetics/iyad159 (PMC10550314; doi:10.1093/genetics/iyad159)
Supplement: iyad159_Supplementary_Data [file iyad159_supplementary_data.zip › Supplemental_Figure_Legends_GENETICS-2023-306198.docx]

**Supplementary figure legends**

**Figure S1. Coiled-coil predictions for budding yeast Smc1 and human SMC1A proteins.** Histograms indicate the probability of coiled-coil formation, calculated using Paircoil2(McDonnell et al. 2006), plotted along the length of human SMC1A and yeast Smc1 proteins.

**Figure S2. A. Conserved CdLS-associated mutations transferred into the yeast *SMC1* gene cause minimal growth defects.** Haploid yeast strains bearing the indicated CdLS-associated mutations in the *SMC1* gene were streaked for single colonies on rich growth medium (YPAD) and incubated at 30°C. **B. Immunoblot analysis of Smc1p expression.** The Smc1 gene in strains in shown in A were tagged at their C termini with an HA epitope tag, and protein expression during mid log phase was assessed by immunoblot analysis. Anti-PGK was used as a loading control.

**Figure S3. Conserved CdLS-associated mutations in the yeast *SMC1* gene result in spindle checkpoint-mediated metaphase delays.** The graph shows the metaphase duration of each individual cell scored for the experiments in Figure 6 D and E. In each column cells from a given replicate are similarly colored. Cells that failed to exit metaphase over the course of the experiment are indicated with a similarly colored X, and the elapsed time in metaphase for these cells was included in the statistical analysis. The number of biological replicates and number of cells scored in each replicate are indicated beneath the graph. Some individual data points are hidden under over-lapping data points.
