# Supplementary material for: Cornelia de Lange Syndrome mutations in SMC1A cause cohesion defects in yeast
Source: Genetics. 2023 Aug 31;225(2):iyad159. doi: 10.1093/genetics/iyad159 (PMC10550314; doi:10.1093/genetics/iyad159)
Supplement: iyad159_Supplementary_Data [file iyad159_supplementary_data.zip › Supplemental_Tables_GENETICS-2023-306198.docx]

**Table S1: Diploid strain list**

| **Strain name** | **Genotype** | **Name in Figure** | **Figure(s)** |
| --- | --- | --- | --- |
| YNN281 | *MAT***a**/α, *ura3-52*/*ura3-52*, *ade2-101*/*ade2-101*, *lys2-801*/*lys2-801*, *trp1-Δ1*/ *trp1-Δ1*, CFIII[*SUP11 URA3 CEN6*] | WT/WT | 4B, 4C |
| DJC3 | *MAT***a**/α, *ura3-52*/*ura3-52*, *ade2-101*/*ade2-101*, *lys2-801*/*lys2-801*, *trp1-Δ1/trp1-Δ1*, *smc1-K511H*/*SMC1*, CFIII[*SUP11 URA3 CEN6*] | K511H | 4B |
| DJC4 | *MAT***a**/α, *ura3-52*/*ura3-52*, *ade2-101*/*ade2-101*, *lys2-801*/*lys2-801*, *trp1-Δ1/trp1-Δ1, smc1-K511H/smc1-K511H,* CFIII[*SUP11 URA3 CEN6*] | K511H | 4C |
| DJC11 | *MAT***a**/α, *ura3-52*/*ura3-52*, *ade2-101*/*ade2-101*, *lys2-801*/*lys2-801*, *trp1-Δ1/trp1-Δ1*, *smc1-K801Q*/*SMC1*, CFIII[*SUP11 URA3 CEN6*] | K801Q | 4B |
| DJC12 | *MAT***a**/α, *ura3-52*/*ura3-52*, *ade2-101*/*ade2-101*, *lys2-801*/*lys2-801*, *trp1-Δ1/trp1-Δ1, smc1-K801Q*/*smc1-K801Q,* CFIII[*SUP11 URA3 CEN6*] | K801Q | 4C |
| DJC15 | *MAT***a**/α, *ura3-52*/*ura3-52*, *ade2-101*/*ade2-101*, *lys2-801*/*lys2-801*, *trp1-Δ1/trp1-Δ1*, *smc1-Δ59-63*/*SMC1*, CFIII[*SUP11 URA3 CEN6*] | Δ59-63 | 4B |
| DJC16 | *MAT***a**/α, *ura3-52*/*ura3-52*, *ade2-101*/*ade2-101*, *lys2-801*/*lys2-801*, *trp1-Δ1/trp1-Δ1, smc1 Δ59-63/ smc1-Δ59-63,* CFIII[*SUP11 URA3 CEN6*] | Δ59-63 | 4C |
| DJC17 | *MAT***a**/α, *ura3-52*/*ura3-52*, *ade2-101*/*ade2-101*, *lys2-801*/*lys2-801*, *trp1-Δ1/trp1-Δ1*, *smc1-F147V*/*SMC1*, CFIII[*SUP11 URA3 CEN6*] | F147V | 4B |
| DJC18 | *MAT***a**/α, *ura3-52*/*ura3-52*, *ade2-101*/*ade2-101*, *lys2-801*/*lys2-801*, *trp1-Δ1/trp1-Δ1, smc1-F147V/smc1-F147V,* CFIII[*SUP11 URA3 CEN6*] | F147V | 4C |
| DJC19 | *MAT***a**/α, *ura3-52*/*ura3-52*, *ade2-101*/*ade2-101*, *lys2-801*/*lys2-801*, *trp1-Δ1/trp1-Δ1*, *smc1-E508A*/*SMC1*, CFIII[*SUP11 URA3 CEN6*] | E508A | 4B |
| DJC20 | *MAT***a**/α, *ura3-52*/*ura3-52*, *ade2-101*/*ade2-101*, *lys2-801*/*lys2-801*, *trp1-Δ1/trp1-Δ1, smc1-E508A/smc1-E508A,* CFIII[*SUP11 URA3 CEN6*] | E508A | 4C |
| DJC21 | *MAT***a**/α, *ura3-52*/*ura3-52*, *ade2-101*/*ade2-101*, *lys2-801*/*lys2-801*, *trp1-Δ1/trp1-Δ1,* *smc1-F1123L*/*SMC1*, CFIII[*SUP11 URA3 CEN6*] | F1123L | 4B |
| DJC22 | *MAT***a**/α, *ura3-52*/*ura3-52*, *ade2-101*/*ade2-101*, *lys2-801*/*lys2-801*, *trp1-Δ1/trp1-Δ1, smc1-F1123L/smc1-F1123L,* CFIII[*SUP11 URA3 CEN6*] | F1123L | 4C |
| SR836 | *MAT***a**/α, *ura3-52*/*ura3-52*, *ade2-101*/*ade2-101*, *lys2-801*/*lys2-801*, *trp1-Δ1/trp1-Δ1*, s*mc1Δ::kanMX6/SMC1*, CFIII[*SUP11 URA3 CEN6*] | WT/- | 4B, 4C |

**Table S2: Haploid strain list**

| **Strain name** | **Genotype** | **Name in Figure** | **Figure(s)** |
| --- | --- | --- | --- |
| O871 | *MAT***a***, ade2-101, lys2-801, trp1-∆1, ura3-52, NET1-GFP-CaURA3* | WT | 3B |
| O882 | *MAT***a***, ade2-101, lys2-801, trp1-∆1, ura3-52, NET1-GFP-CaURA3, smc1-K511H* | K511H | 3B |
| O893 | *MAT***a***, ade2-101, lys2-801, trp1-∆1, ura3-52, NET1-GFP-CaURA3, smc1-E508A* | E508A | 3B |
| O897 | *MAT***a***, ade2-101, lys2-801, trp1-∆1, ura3-52, NET1-GFP-CaURA3, smc1-F147V* | F147V | 3B |
| O899 | *MAT***a***, ade2-101, lys2-801, trp1-∆1, ura3-52, NET1-GFP-CaURA3, smc1-F1123L* | F1123L | 3B |
| O901 | *MAT***a***, ade2-101, lys2-801, trp1-∆1, ura3-52, NET1-GFP-CaURA3, smc1-Δ59-63* | Δ59-63 | 3B |
| O911 | *MAT***a***, ade2-101, lys2-801, trp1-∆1, ura3-52, NET1-GFP-CaURA3, smc1-K801Q* | K801Q | 3B |
| O951 | *MAT***a***, ade2-101, lys2-801, trp1-Δ1, ura3-52, smc1-K511H* | K511H | 3C, S2 |
| O953 | *MAT***a***, ade2-101, lys2-801, trp1-Δ1, ura3-52, smc1-K801Q* | K801Q | 3C, S2 |
| O954 | *MAT***a***, ade2-101, lys2-801, trp1-Δ1, ura3-52, smc1-Δ59-63* | Δ59-63 | 3C, S2 |
| O955 | *MAT***a***, ade2-101, lys2-801, trp1-Δ1, ura3-52, smc1-F147V* | F147V | 3C, S2 |
| O956 | *MAT***a***, ade2-101, lys2-801, trp1-Δ1, ura3-52, smc1-E508A* | E508A | 3C, S2 |
| O957 | *MAT***a***, ade2-101, lys2-801, trp1-Δ1, ura3-52, smc1-F1123L* | F1123L | 3C, S2 |
| O958 | *MAT***a***, ade2-101, lys2-801, trp1-Δ1, ura3-52, SMC1* | WT | 3C, S2 |
| O959 | *MAT***a***, ade2-101, lys2-801, trp1-Δ1, ura3-52, smc1-K511H, SPC42-yoTagRFP-T-Kan* | K511H | 6D, S3 |
| O961 | *MAT***a***, ade2-101, lys2-801, trp1-Δ1, ura3-52, smc1-K801Q, SPC42-yoTagRFP-T-Kan* | K801Q | 6D, S3 |
| O962 | *MAT***a***, ade2-101, lys2-801, trp1-Δ1, ura3-52, smc1-Δ59-63, SPC42-yoTagRFP-T-Kan* | Δ59-63 | 6C, 6D, S3 |
| O963 | *MAT***a***, ade2-101, lys2-801, trp1-Δ1, ura3-52, smc1-F147V, SPC42-yoTagRFP-T-Kan* | F147V | 6D, S3 |
| O964 | *MAT***a***, ade2-101, lys2-801, trp1-Δ1, ura3-52, smc1-E508A, SPC42-yoTagRFP-T-Kan* | E508A | 6D, S3 |
| O965 | *MAT***a***, ade2-101, lys2-801, trp1-Δ1, ura3-52, smc1-F1123L, SPC42-yoTagRFP-T-Kan* | F1123L | 6D, S3 |
| O1000 | *MAT***a***, ade2-101, lys2-801, trp1-Δ1, ura3-52, SMC1, SPC42-yoTagRFP-T-Kan* | WT | 6C, 6D, S3 |
| O1057 | *MAT***a***, ade2-101, lys2-801, trp1-Δ1, ura3-52::pDO2[URA3 P_CYC1_-GFP-lacI], SMC1, SPC42-yoTagRFP-T-Kan, CEN3::pJC287[lacO ADE2]* | WT | 5B |
| O1059 | *MAT***a***, ade2-101, lys2-801, trp1-Δ1, ura3-52::pDO2[URA3 P_CYC1_-GFP-lacI], smc1-K511H, SPC42-yoTagRFP-T-Kan, CEN3::pJC287[lacO ADE2]* | K51H | 5B |
| O1062 | *MAT***a***, ade2-101, lys2-801, trp1-Δ1, ura3-52::pDO2[URA3 P_CYC1_-GFP-lacI], smc1-K801Q, SPC42-yoTagRFP-T-Kan, CEN3::pJC287[lacO ADE2]* | K801Q | 5B |
| O1063 | *MAT***a***, ade2-101, lys2-801, trp1-Δ1, ura3-52::pDO2[URA3 P_CYC1_-GFP-lacI], smc1-Δ59-63, SPC42-yoTagRFP-T-Kan, CEN3::pJC287[lacO ADE2]* | Δ59-63 | 5B |
| O1064 | *MAT***a***, ade2-101, lys2-801, trp1-Δ1, ura3-52::pDO2[URA3 P_CYC1_-GFP-lacI], smc1-F147V, SPC42-yoTagRFP-T-Kan, CEN3::pJC287[lacO ADE2]* | F147V | 5B |
| O1065 | *MAT***a***, ade2-101, lys2-801, trp1-Δ1, ura3-52::pDO2[URA3 P_CYC1_-GFP-lacI], smc1-E508A, SPC42-yoTagRFP-T-Kan, CEN3::pJC287[lacO ADE2]* | E508A | 5B |
| O1066 | *MAT***a***, ade2-101, lys2-801, trp1-Δ1, ura3-52::pDO2[URA3 P_CYC1_-GFP-lacI], smc1-F1123L, SPC42-yoTagRFP-T-Kan, CEN3::pJC287[lacO ADE2]* | F1123L | 5B |
| O1077 | *MAT***a***, ade2-101, lys2-801, trp1-Δ1, ura3-52, SMC1, SPC42-yoTagRFP-T-Kan, mad2::natNT2* | WT | 6E, S3 |
| O1078 | *MAT***a***, ade2-101, lys2-801, trp1-Δ1, ura3-52, smc1-K511H, SPC42-yoTagRFP-T-Kan, mad2::natNT2* | K511H | 6E, S3 |
| O1079 | *MAT***a***, ade2-101, lys2-801, trp1-Δ1, ura3-52, smc1-K801Q, SPC42-yoTagRFP-T-Kan, mad2::natNT2* | K801Q | 6E, S3 |
| O1082 | *MAT***a***, ade2-101, lys2-801, trp1-Δ1, ura3-52, smc1-Δ59-63, SPC42-yoTagRFP-T-Kan, mad2::natNT2* | Δ59-63 | 6E, S3 |
| O1083 | *MAT***a***, ade2-101, lys2-801, trp1-Δ1, ura3-52, smc1-F147V, SPC42-yoTagRFP-T-Kan, mad2::natNT2* | F147V | 6E, S3 |
| O1084 | *MAT***a***, ade2-101, lys2-801, trp1-Δ1, ura3-52, smc1-E508A, SPC42-yoTagRFP-T-Kan, mad2::natNT2* | E508A | 6E, S3 |
| O1085 | *MAT***a***, ade2-101, lys2-801, trp1-Δ1, ura3-52, smc1-F1123L, SPC42-yoTagRFP-T-Kan, mad2::natNT2* | F1123L | 6E, S3 |
| O1137 | *MAT***a***, ade2-101, lys2-801, trp1-Δ1, ura3-52, smc1-K511H-6HA-KANMX4* | K511H |  |
| O1138 | *MAT***a***, ade2-101, lys2-801, trp1-Δ1, ura3-52, smc1-K801Q-6HA-KANMX4* | K801Q |  |
| O1139 | *MAT***a***, ade2-101, lys2-801, trp1-Δ1, ura3-52, smc1-Δ59-63-6HA-KANMX4* | Δ59-63 |  |
| O1140 | *MAT***a***, ade2-101, lys2-801, trp1-Δ1, ura3-52, smc1-F147V-6HA-KANMX4* | F147V |  |
| O1141 | *MAT***a***, ade2-101, lys2-801, trp1-Δ1, ura3-52, smc1-E508A-6HA-KANMX4* | E508A |  |
| O1142 | *MAT***a***, ade2-101, lys2-801, trp1-Δ1, ura3-52, smc1-F1123L-6HA-KANMX4* | F1123L |  |
| O1143 | *MAT***a***, ade2-101, lys2-801, trp1-Δ1, ura3-52, SMC1-6HA-KANMX4* | WT |  |

**Table S3: Primers to construct the donor plasmids**

| Mutation | Primer | Nucleotide sequences (5’-3’) |
| --- | --- | --- |
| Common primers | SR271 | TATAGGGCGAATTGGGTACCGTGGAGAGATTTGAAACACAACTAAAAGTGG |
|  | SR286 | GGGAACAAAAGCTGGAGCTCATATCTTCCTTTATACCGTCTCTTTCCC |
|  | SR263 | TATAGGGCGAATTGGGTACCCCGTGGGCAGAAGAAATACA |
|  | SR270 | GGGAACAAAAGCTGGAGCTCGGATTGACCCACCTTCAGTCAGATA |
| K511H | SR274 | GCTAATCAAAGAGAAACAATGCACGAAAGAAAACTAAG |
|  | SR275 | CGTGCATTGTTTCTCTTTGATTAGCACTCAAGTC |
| K801Q | SR282 | GCTTTACAATCCAAGAATATGAAAATCATTCCGGTG |
|  | SR283 | GATTTTCATATTCTTGGATTGTAAAGCCAATTTTACTAG |
| Δ59-63 | SR264 | GTAATCATTTGAGGGATTTAATCTATAGAGGTGTTC |
|  | SR265 | CTCTATAGATTAAATCCCTCAAATGATTACTCCGC |
| F147V | SR266 | AAGCCAAAAATGTTCTAGTGTTCCAGGGTG |
|  | SR267 | CCTGGAACACTAGAACATTTTTGGCTTTGATAAGAATATTTT |
| E508A | SR272 | GCTAATCAAAGAGCAACAATGAAAGAAAGAAAACTAAG |
|  | SR273 | CTTTCATTGTTGCTCTTTGATTAGCACTCAAGTC |
| F1123L | SR287 | TATAGGGCGAATTGGGTACCCCAGCGAAGATATTCTAGAGGAC |
|  | SR288 | GCCTCTTAAAAGACTCAAAGACATGGAATAT |
|  | SR289 | GTCTTTGAGTCTTTTAAGAGGCGGAG |
|  | SR290 | GGGAACAAAAGCTGGAGCTCCGGGTTATAGCAGAGGTTGG |
| ΔSMC1 | SR610 | AAAGCAAGCATCCAGAGGCTATTGATAAAAAGCAGGCACAAGGAGACGCAcggatccccgggttaattaa |
|  | SR611 | TAGATATTATTAGTTATTTGACGGGTTATAGCAGAGGTTGGTTTCATAGAgaattcgagctcgtttaaac |
| SMC1 | OPR1273 | GTTATTAATATTATTGTTACTATGATATTGCACTAGCTAGTTGACCTATAatcgatgaattcgagctcg |
|  | OPR1274 | TCTATGAAACCAACCTCTGCTATAACCCGTCAAATAACTAATAATATCTAcgtacgctgcaggtcgac |
| SPC42 | OPR1435 | CTGAAAATAATATGTCAGAAACATTCGCAACTCCCACTCCCAATAATCGAggtgacggtgctggttta |
|  | OPR1436 | AGAACGCTTTAAGAATGCGCCATACTCCTTAACTGCTTTTTAAATCATCAtcgatgaattcgagctcg |
| ADE2 | OPR1444 | tactcaggtatcgtaagatgcaagagttcgttcttgaataatacataacttttcttaaaa |
|  | OPR1445 | tctattatgaatttcatttataaagtttatgatcttatgtatgaaattcttaaaaaagga |
| ΔMAD2 | OPR317 | CGCCCCGAAGAATGATTGATGTTAAATACTCGTACAAGAGTATTGAAAACCgtacgctgcaggtcgac |
|  | OPR318 | CGCATGAAGTGGTTTACAGAGGAAGTACGTAGTATAGTATAATATAGTTCAtcgatgaattcgagctcg |
| 6HA Tag | OPR1512 | TAGATATTATTAGTTATTTGACGGGTTATAGCAGAGGTTGGTTTCATAGAatcgatgaattcgagctcg |
|  | OPR1513 | AAGAAAACTCGTCGAAGATCATAACTTTGGACTTGAGCAATTACGCAGAAcgtacgctgcaggtcgac |

**Table S4: Primers for mutation confirmation**

| Mutation | Primer | Nucleotide sequences (5’-3’) |
| --- | --- | --- |
| K511H | SR309 | ATCAAAGAGAAACAATGCAC |
|  | SR311 | ATCAAAGAGAAACAATGAAA |
| K801Q | SR316 | AGTAAAATTGGCTTTACAATCC |
|  | SR317 | AGTAAAATTGGCTTTACAATCA |
| Δ59-63 | SR301 | TCATTTGAGGGATTTAATCT |
|  | SR302 | TCATTTGAGGTCAAACATCT |
| F147V | SR303 | TCTTATCAAAGCCAAAAATG |
|  | SR304 | TCTTATCAAAGCCAAAAATT |
| E508A | SR307 | TTGAGTGCTAATCAAAGAGC |
|  | SR308 | TTGAGTGCTAATCAAAGAGA |
| F1123L | SR320 | CACTCCGCCTCTTAAAAGAC |
|  | SR321 | CACTCCGCCTCTTAAAAGAT |
| ΔSMC1 | SR612 | cacatctcataaaatcactt |
|  | SR613 | cactagctagttgacctata |
| SPC42 | OPR1258 | AGTACTGACAATAAAAAGAT |
|  | OPR184 | TGTTGGAAAATAACAATGGA |
| ADE2 | OPR1447 | aacgctgcgctgttggctgt |
|  | OPR1448 | atttagttcttaataaaagg |
| ΔMAD2 | OPR417 | TAGGTATATGTGGCTCAAAAATG |
|  | OPR423 | AGAAACAGACGACACTGACG |
| 6HA Tag | OPR1066 | ATACTGCTGTCGATTCGATACT |
|  | OPR957 | cactagctagttgacctata |
