# Supplementary figures and images for: Cornelia de Lange Syndrome mutations in SMC1A cause cohesion defects in yeast
Source: Genetics. 2023 Aug 31;225(2):iyad159. doi: 10.1093/genetics/iyad159 (PMC10550314; doi:10.1093/genetics/iyad159)

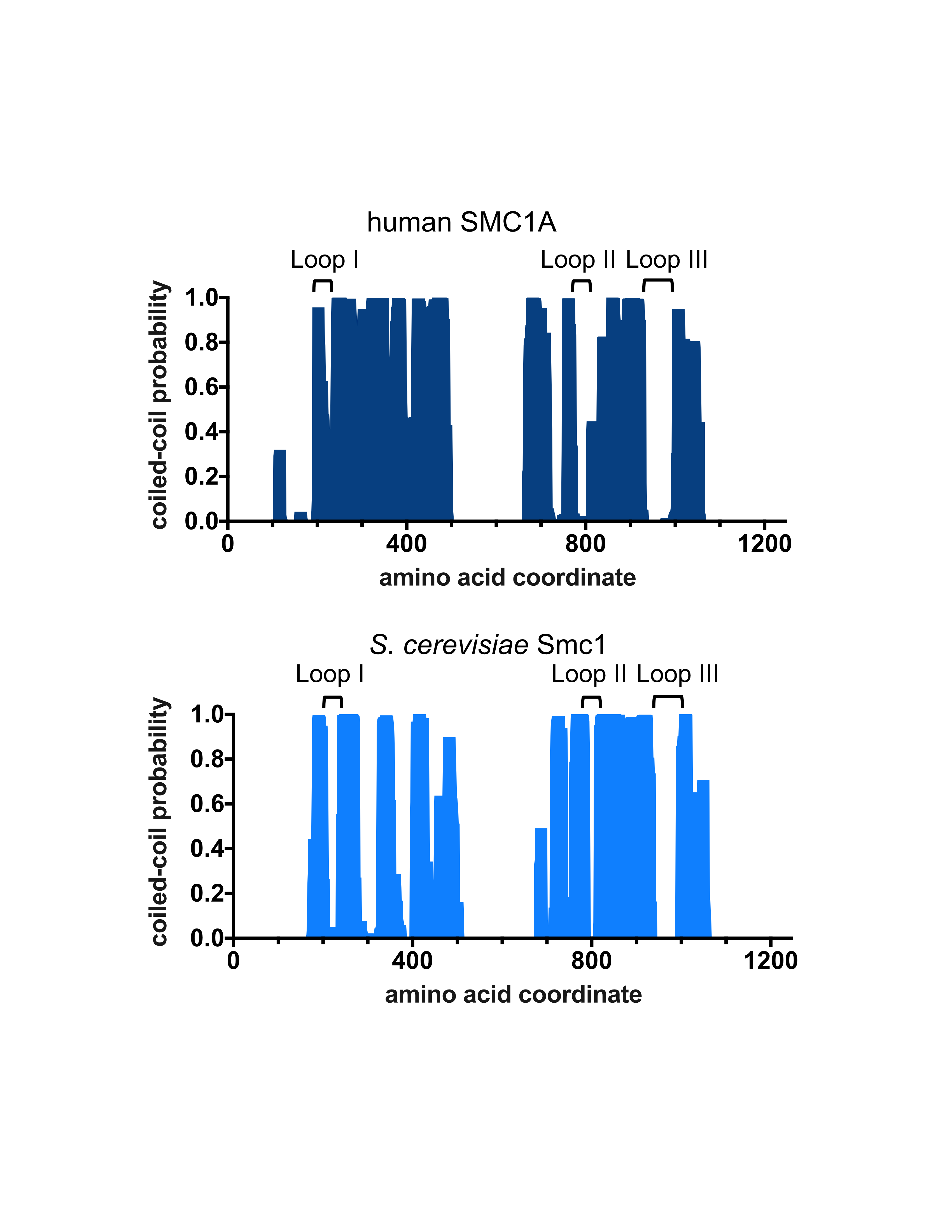

Supplement: iyad159_Supplementary_Data [file iyad159_supplementary_data.zip › Figure_S1_GENETICS-2023-306198.tif]

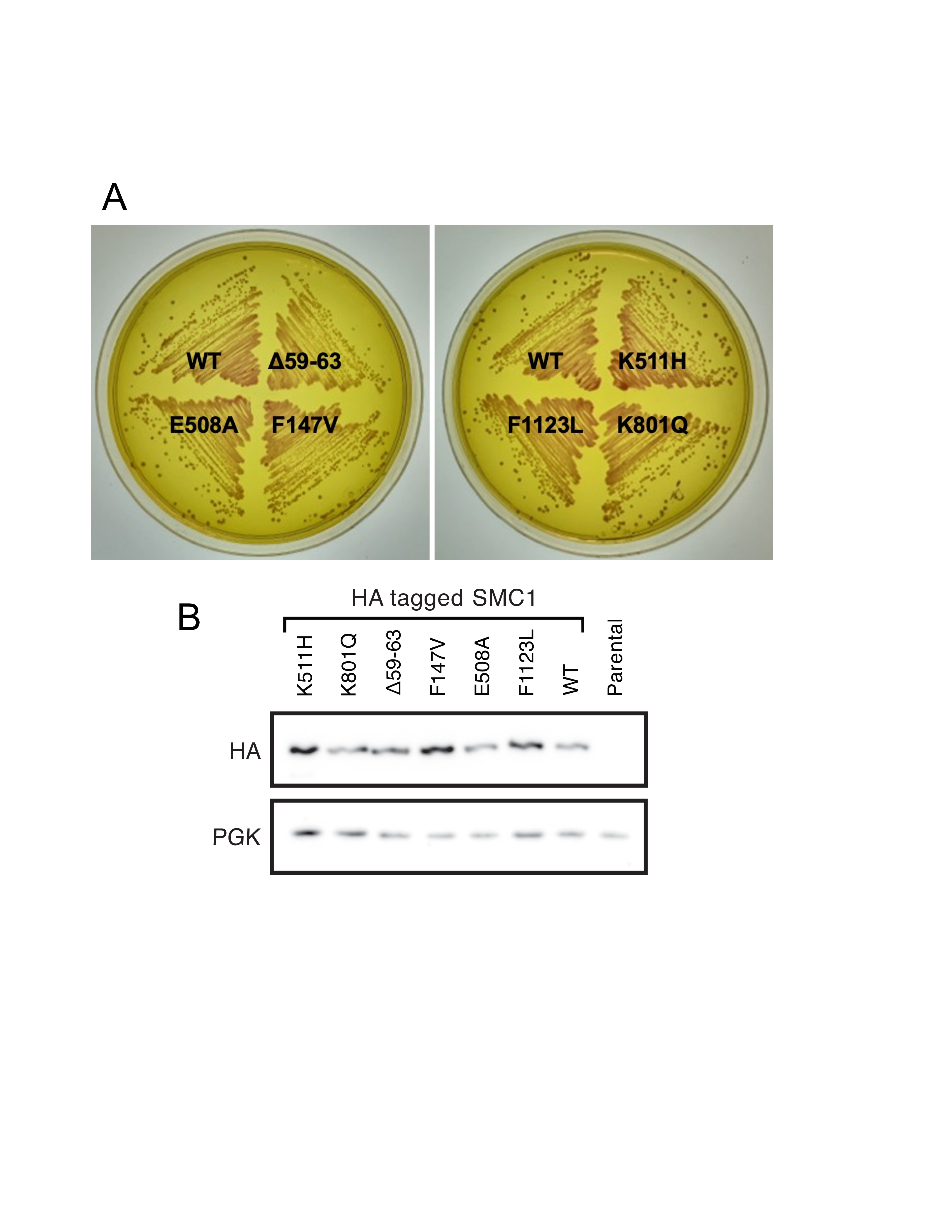

Supplement: iyad159_Supplementary_Data [file iyad159_supplementary_data.zip › Figure_S2_GENETICS-2023-306198.tif]

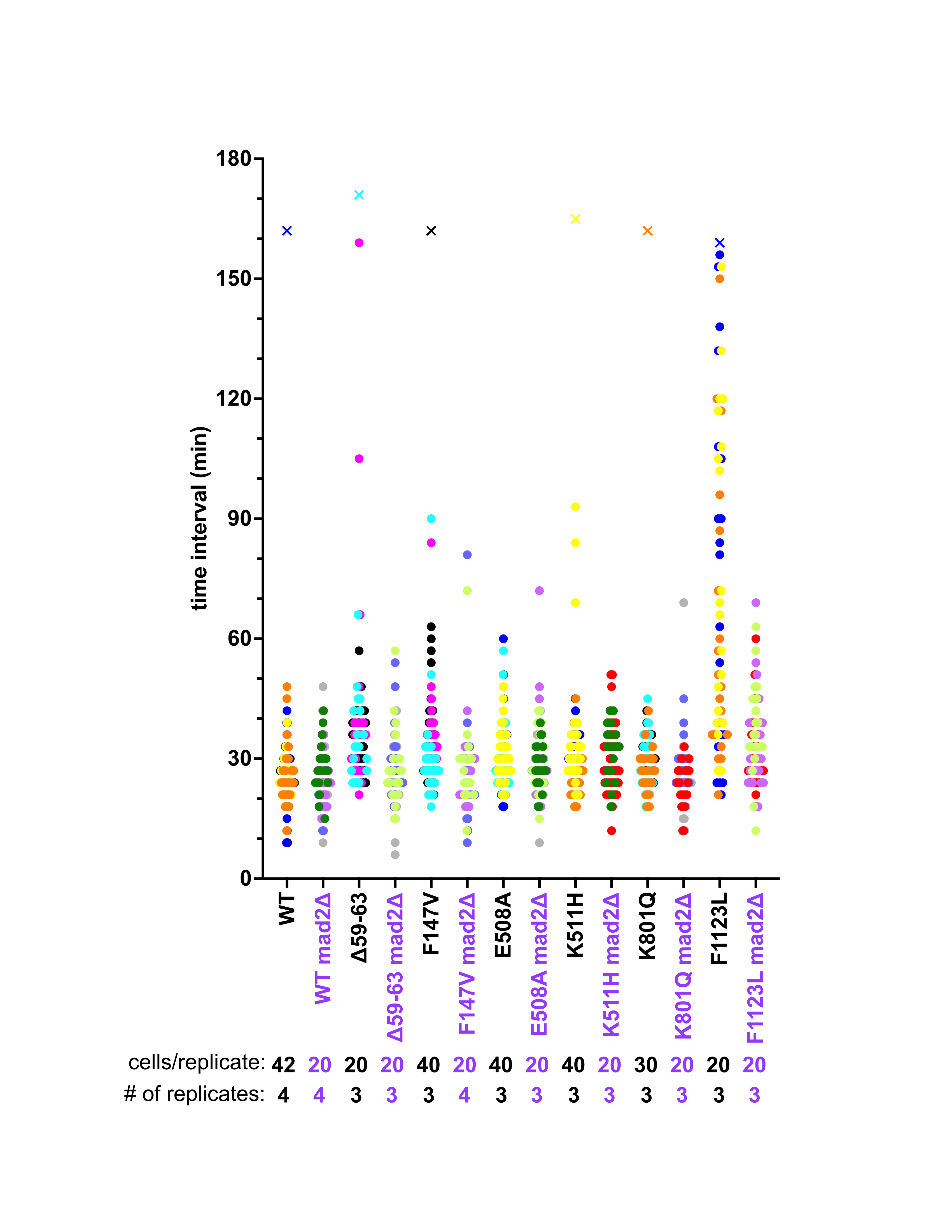

Supplement: iyad159_Supplementary_Data [file iyad159_supplementary_data.zip › Figure_S3_GENETICS-2023-306198.tif]
